# 

**Published:** 1915-08-07

**Authors:** 


Colonel Thomas Wilson, C.B., of Liverpool, who left estate valued at £138,167 gross, has bequeathed £100 each to the Royal Infirmary, Liverpool; the Liverpool Royal Southern Hospital; the David Lewis Royal Northern Hospital; and the Hospital for Children, Catherine Street, Liverpool.

RATES OF SUBSCRIPTION: Twelve months, 6/6; Six months, 4/-; Three months, 2/-, post free to any address in the United Kingdom. Abroad, Twelve months, 8/8; Six months, 5/-; Three months, 2/6, post free.—Address The Subscription Dept., "THE HOSPITAL," The Hospital Building, 28/29 Southampton Street, Strand, London, W.C.

